# [Retracted] Isoquercitrin inhibits bladder cancer progression *in vitro* and *in vivo* by regulating the PI3K/Akt and PKC signaling pathways

**DOI:** 10.3892/or.2023.8684

**Published:** 2023-12-18

**Authors:** Feng Chen, Xiaochi Chen, Deyong Yang, Xiangyu Che, Jianbo Wang, Xiancheng Li, Zhiwei Zhang, Qifei Wang, Wei Zheng, Lina Wang, Xuejian Wang, Xishuang Song

Oncol Rep 36: 165–172, 2016; DOI: 10.3892/or.2016.4794

Following the publication of this paper, it was drawn to the Editor's attention by a concerned reader that the agarose gel electrophoretic bands shown in Fig. 4A for PKC were strikingly similar to bands that had already appeared in another article written by different authors at different research institutes.

Owing to the fact that the contentious data in the above article had already been published prior to its submission to Oncology Reports, the Editor has decided that this paper should be retracted from the Journal. The authors were asked for an explanation to account for these concerns, but the Editorial Office did not receive a reply. The Editor apologizes to the readership for any inconvenience caused.

